# Cortical firing dynamics during micro-arousals vary with sleep/wake history and micro-arousal duration

**DOI:** 10.1038/s41598-026-45192-y

**Published:** 2026-04-01

**Authors:** Natalie L. Hauglund, Lukas B. Krone, Martin Kahn, Cristina Blanco-Duque, Vladyslav V. Vyazovskiy

**Affiliations:** 1 Department of Physiology, Anatomy and Genetics, University of Oxford, Oxford, OX1 3PT UK; 2 Department of Clinical Neurophysiology, Danish Center for Sleep Medicine, 2600 Rigshospitalet, Glostrup, Denmark; 3 Sleep and Circadian Neuroscience Institute, University of Oxford, Oxford, OX1 3QU UK; 4 Kavli Institute of Nanoscience Discovery, University of Oxford, Sherrington Rd, Oxford, OX1 3QU UK; 5 Centre for Neural Circuits and Behaviour, University of Oxford, Oxford, OX1 3SR UK; 6 University Hospital of Psychiatry and Psychotherapy, University of Bern, Bern, Switzerland; 7 Picower Institute for Learning and Memory, Massachusetts Institute of Technology, Cambridge, MA 02139 USA

**Keywords:** Neuroscience, Circadian rhythms and sleep

## Abstract

Non-rapid eye movement (NREM) sleep is not uniform but characterized by brief intrusions of wake-like brain activity and increased muscle tonus known as micro-arousals or brief awakenings. Although micro-arousals are an inherent feature of human and animal sleep, knowledge about the neural correlates of micro-arousals is sparse. We here developed an algorithm for automatic detection of micro-arousals based on EMG activity and used it to analyse the associated laminar neural activity in the motor cortex in mice. Our analysis showed that short micro-arousals with a duration below 5 s were associated with decreased cortical firing, while longer micro-arousals with a duration of 5–10 s were associated with increased firing in a similar manner to transitions to wakefulness. Analysis of single-channel firing showed that some channels exhibited increased activity immediately prior to micro-arousals, while others exhibited decreased activity. Slow wave activity (SWA, 1–4 Hz) immediately after micro-arousals was tightly correlated with sleep pressure and even surpassed average SWA levels during NREM sleep in sleep deprived animals. This study provides new insights into the neural mechanisms associated with micro-arousals and identifies a new link between sleep architecture and sleep homeostasis.

**Supplementary Information:**

The online version contains supplementary material available at 10.1038/s41598-026-45192-y.

## Introduction

The restorative effect of sleep is traditionally considered to rely on long and uninterrupted sleep periods. However, non-rapid eye movement (NREM) sleep is interspersed by regular arousals characterised by a brief shift of the electroencephalogram (EEG) frequencies followed by transition directly back to NREM sleep. These “micro-arousals” are normally not consciously perceived, occur even in the absence of external stimuli, and their functional significance is not known^1,2^.

Arousals from sleep have had several criteria and definitions over the years. The first manual for standardized terminology by Rechtschaffen and Kales in 1968 mentions “movement arousals” defined as an increase in EMG accompanied by a change in the EEG, including a decrease in amplitude, an increase in alpha activity, or a burst of high voltage activity^3^. A few years later in 1971, Schieber et al.^4^ describe arousals from sleep without a full awakening as “phases of spontaneous transitory activation” (PAT or “les phases d’activation transitoire spontanees”), which they define based on a transitory change in EEG and muscle activity. In 1979, Halász and colleagues use the term “micro-arousals” but base the definition on cortical activity patterns rather than muscle activity as they define micro-arousals as K-complexes with or without subsequent muscle activity and EEG synchronization^1,5^. Later, the term micro-arousal has also been used by other groups to define arousals from sleep based primarily on muscle activity and EEG desynchronization^6–8^. In parallel, “brief awakenings” and “spontaneous arousals” are similarly used to define short arousals from sleep based on the presence of muscle activity and/or wake-like EEG^9–11^. Despite this plethora of descriptions and definitions, they likely account for the same phenomenon; repeated activations of the arousal system thought to arise from the internally-generated infraslow oscillation in noradrenaline (NA) released from the brain stem nucleus locus coeruleus (LC) during NREM sleep^12–15^. Studies in mice have shown that the amplitude of the NA surge predicts the behavioural outcome. Thus, a small increase in NA is more likely to result in a purely cortical shift in EEG frequencies displayed by a brief increase in the amplitude of low (< 4 Hz) frequencies and no muscle activity, a larger amplitude NA surge is more likely to result in a desynchronization of the EEG and brief muscle activity, and the largest amplitude NA surges are more often followed by a long period of wakefulness^13,16^. We will here use the term “micro-arousal” and base the definition on a transitory increase in muscle activity with a duration below 10 s. Thus, we will only consider EMG-defined micro-arousals and not purely cortical events.

A wide range of theories on the function of micro-arousals have been proposed. Some suggest that they have a homeostatic function to maintain or restore physiological processes, such as preventing that sleep becomes too deep^17^, preventing premature entry to REM sleep^17^, or to restore cardiorespiratory function in sleep^18^. Another theory is that micro-arousals provide an opportunity to scan the environment for potential dangers without disrupting sleep^1^. Recent studies showed that infraslow cycles in activity of the LC during sleep is implicated in memory formation^13^ and drives cerebrospinal fluid flow, and thereby cleaning of the brain via the glymphatic system during sleep^8^. Thus, the mechanisms responsible for generating micro-arousals have a crucial role in the restorative processes that take place during sleep^2^.

The number of awakenings from sleep exhibit a strong negative correlation with self-reported sleep quality^19^. However, many studies do not clearly distinguish between full awakenings, followed by an extended period of wakefulness, and micro-arousals where sleep is resumed after a few seconds, which makes it difficult to assess the association between internally-generated micro-arousals and subjective sleep quality or pathology. Factors such as stress have been shown to increase the quantity of micro-arousals due to an increase in the frequency of NA oscillations^14,16^. Similarly, old age is associated with an increase in micro-arousals^20–22^, suggesting that an elevated micro-arousal frequency is indicative of poor sleep. This association between micro-arousals and poor sleep quality has contributed to the common misconception that all micro-arousals per se are equivalent to sleep fragmentation and poor sleep. The relationship between sleep arousals and all-cause mortality is a bell-shaped curve where both a very low number and a high number of micro-arousals are associated with higher mortality rate^23^. Thus, the relationship between micro-arousals and sleep quality is more complicated than “the fewer the better”, which underscores the importance of gaining a better understanding of the interplay between micro-arousals and sleep physiology.

Sleep is under homeostatic regulation and the amplitude of EEG slow wave activity (SWA, typically defined as EEG oscillations between 0.5 and 4 Hz) during NREM sleep increases as a function of time spent awake prior to the sleep episode^24–26^. The generation of micro-arousals is also homeostatically regulated as high sleep pressure is linked to fewer micro-arousals in both humans^27^ and rodents^28,29^. However, it is currently not known whether neuronal activity associated with micro-arousals is affected by sleep pressure.

Micro-arousals are brief events with durations that often falls below four seconds, which is the bin size typically used for rodent sleep scoring. This makes accurate analysis of neuronal activity around micro-arousals difficult. To overcome these challenges, we here developed an algorithm to automatically detect NREM sleep micro-arousals based on the electromyography (EMG) signal. We subsequently used the algorithm to detect micro-arousals in continuous sleep data from mice with laminar local field potential (LFP) and multiunit activity (MUA) recordings from the motor cortex. Recordings from the mice during 24 h of baseline as well as after 6 h of sleep deprivation allowed us to investigate cortical neuronal activity across the motor cortex during micro-arousals, and assess how these activity patterns are impacted by sleep pressure.

## Results

### Automated algorithm for detection of NREM micro-arousals is a useful tool for objective micro-arousal detection

**Fig. 1:**
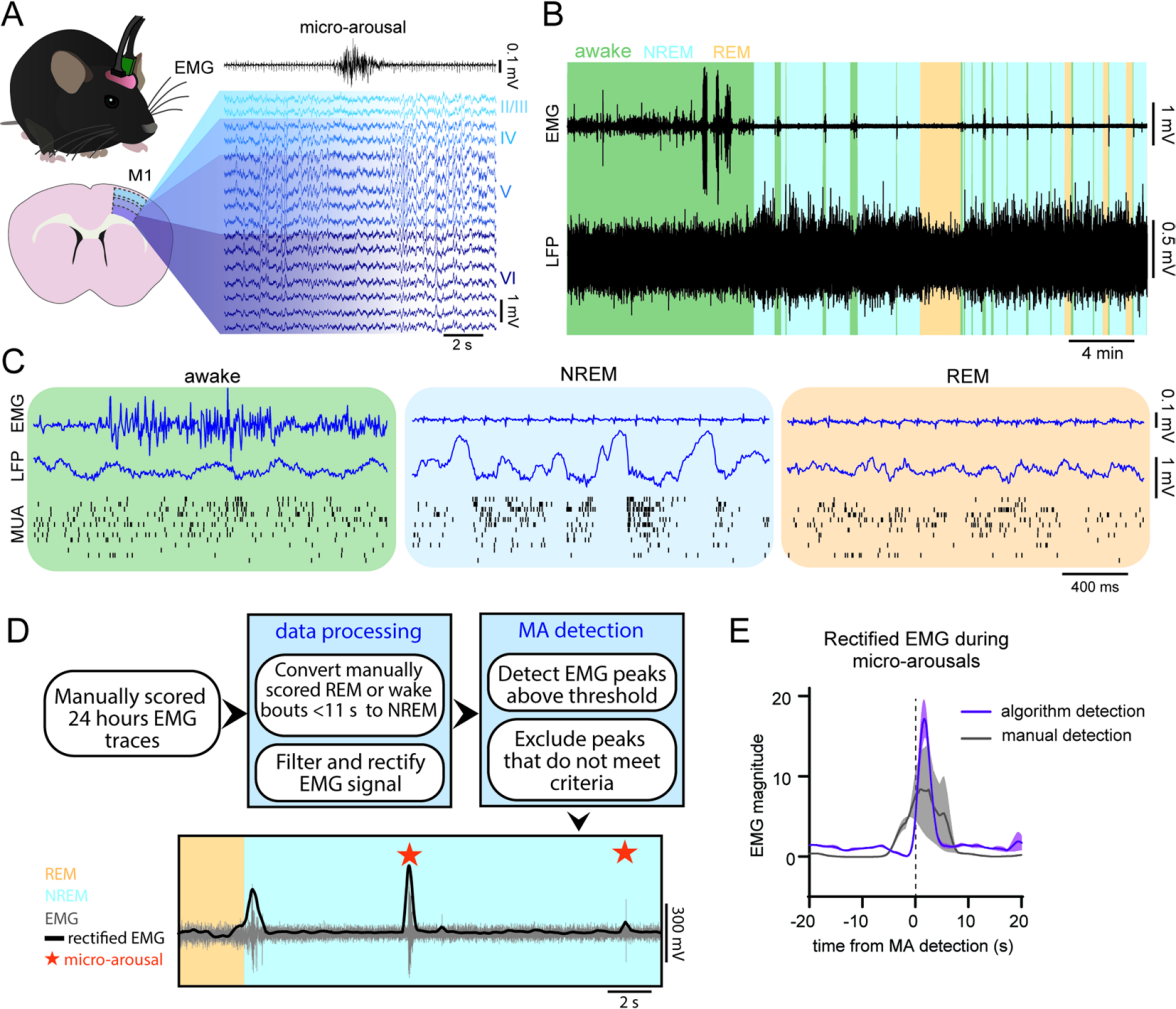
Automated algorithm for detection of NREM micro-arousals is a useful tool for objective micro-arousal detection. (**A**) Diagram of the experimental setup. EMG, EEG and LFP traces from the motor cortex were acquired from C57Bl/6 mice. Micro-arousals were detected by the algorithm and the LFP traces around every micro-arousal were analysed. (**B**) Example EMG and LFP traces with the background colour coded to show vigilance states. (**C**) Example EMG, LFP and multi-unit activity data from wakefulness, NREM sleep and REM sleep. (**D**) The sequential steps of the pipeline for automated detection of micro-arousals. The pipeline is used on EMG data pre-scored for vigilance states. In the first steps, all manually scored wake- or REM sleep bouts with a duration below 11 s within a NREM episode are rescored as NREM sleep to omit subjectively scored micro-arousals. The EMG signal is then filtered and rectified, and EMG peaks during NREM sleep are detected. During the next steps, EMG peaks that do not occur during NREM sleep are excluded and peaks immediately following each other are combined. Finally, EMG peaks that follow REM sleep and EMG peaks with a duration above 10 s are excluded. The last image is an example of the resulting scoring showing the original EMG signal in grey, the rectified EMG used for peak detection in black and the detected micro-arousals as orange stars. Manually scored behavioural states showed as shading in the background. Notice how micro-arousals following REM sleep are not included. (**E**) Mean rectified EMG trace during micro-arousals (MAs) in comparison with algorithm-based and manual detection (n = 7, shaded area is SEM).

In order to precisely assess the neural correlates of micro-arousals, we aimed to detect micro-arousals and their start and end times with high temporal accuracy. We therefore developed an algorithm that detects micro-arousals during NREM sleep based on analysis of the EMG. The micro-arousal detection algorithm was applied to a dataset previously published^30–32^. In this dataset, adult wild-type (C57BL/6) mice were implanted with electrodes for recording laminar LFP and MUA from the left primary motor cortex (M1), concomitantly with EEG and EMG recordings (Fig. 1A). 24 h of baseline data was manually scored as wakefulness, NREM sleep and REM sleep based on the EEG and EMG signal, and LFP and MUA was extracted for analysis (Fig. 1B, C).

While several definitions of micro-arousals exist^1,4,8,13,14,16^, we here defined a micro-arousal as a brief (below 10 s) bout of muscle activity during NREM sleep, based on analysis of the distribution of arousal durations in our data set, which revealed that the vast majority of the events fall below the 10 s duration threshold (see below). The algorithm was developed to work as a post-processing tool on manually-scored sleep data that had already been segmented into the three main vigilance states: wakefulness, NREM sleep and REM sleep. This allowed us to use the algorithm on already obtained datasets in order to re-analyse them with a focus on micro-arousals. Only the EMG signal was used for the detection to allow investigation of the neuronal dynamics in an unbiased manner. The processing pipeline for the algorithm is shown in Fig. 1D. After inputting the EMG trace and the corresponding vigilance state scoring, the EMG was pre-processed and EMG activity peaks were included or excluded based on criteria, such as duration and amplitude (see detailed description in method section). The resulting EMG peaks represented NREM micro-arousals that were used for analysis. An average of the rectified EMG signal during the detected micro-arousals confirmed that the algorithm successfully identified brief increases in EMG tonus (Fig. 1E).

### Short micro-arousals are associated with decreased cortical firing

**Fig. 2:**
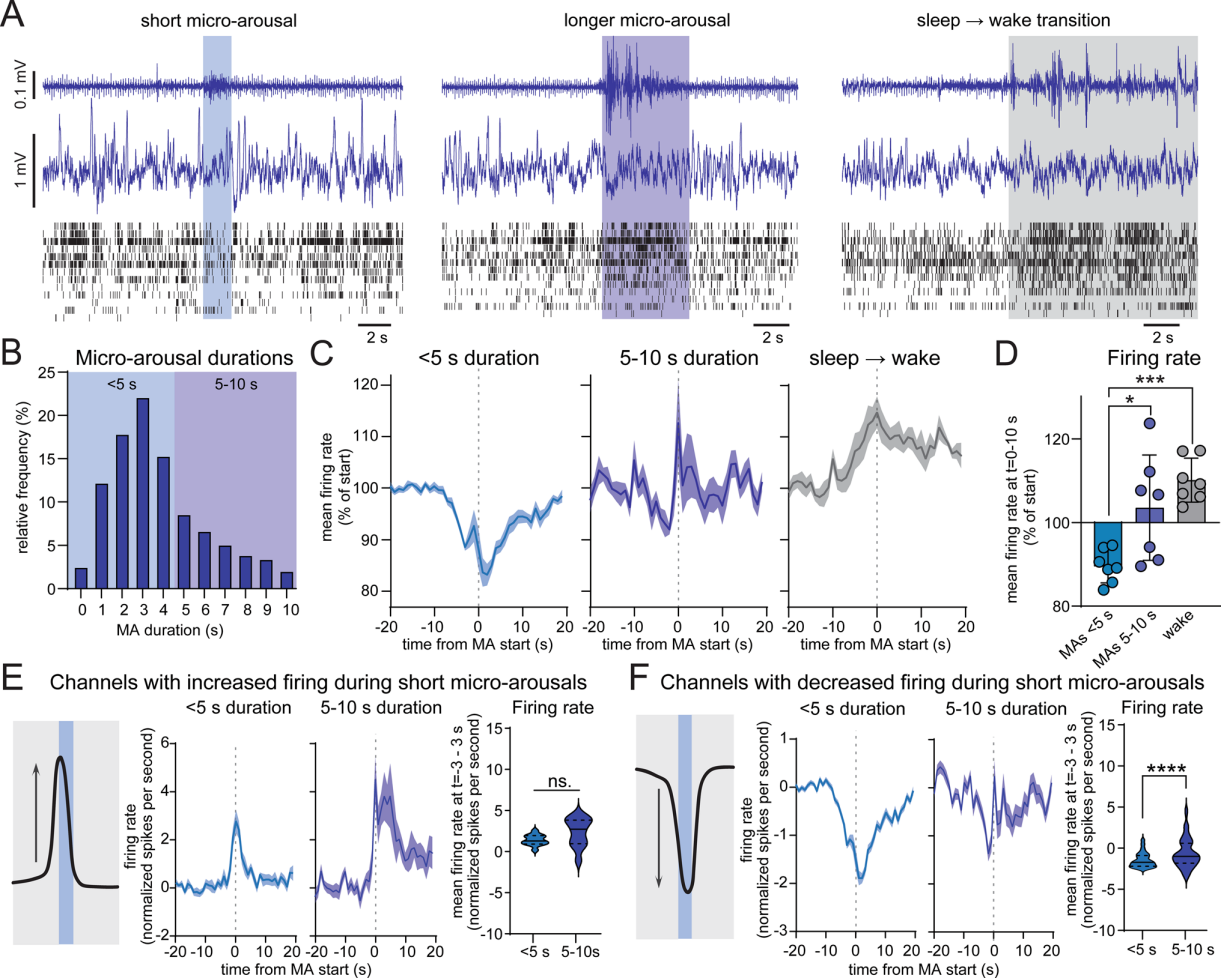
Short micro-arousals are associated with decreased cortical firing. (**A**) Representative EMG, LFP and multi-unit activity from the motor cortex during a short micro-arousal with decreased neuronal firing (left), a longer micro-arousal with increased neuronal firing (middle), and a transition from sleep to wakefulness (right). (**B**) Histogram showing the distribution of micro-arousal durations. 3029 micro-arousals from 7 mice were analysed. The two tones of blue depict the separation of micro-arousals into groups with durations below 5 s and with durations from 5 to 10 s. (**C**) Average number of spikes per second as percent from the firing rate from time − 10 to − 20 during micro-arousals with a duration below 5 s (left), between 5–10 s (middle) and during transitions from sleep to wakefulness (right, wakefulness defined as muscle activity for more than 20 s) (n = 7). (**D**) Average firing rate as percent change from 10 to 20 s before the micro-arousal or wake transition (n = 7, one way ANOVA, *P* = 0.012 for 5 < vs. 5–10 and *P* = 0.0005 for 5 < vs. wake). (**E**) Average firing rate of channels with increased firing during short (left) and longer (right) micro-arousals calculated as spikes per second normalized to the firing rate and standard deviation during the first 10 s of the trace (number of channels = 12). Right panel: quantification of the average firing rate 3 s before to 3 s after the first muscle activity (paired* t* test, dashed lines depict median and interquartile range, *P* = 0.067). (**F**) firing rate of channels with decreased firing during short (left) and longer (right) micro-arousals calculated as spikes per second normalized to the firing rate and standard deviation during the first 10 s of the trace (number of channels = 89). To the right: quantification of the average firing rate 3 s before to 3 s after the first muscle activity (paired *t* test, dashed lines depict median and interquartile range, *P* < 0.0001). Shaded area around line graphs are ± SEM. MA = micro-arousal.

Before applying the automatic micro-arousal detection, the data was inspected visually. From the raw multi-unit activity traces spanning layers 2/3 to layer 6 of the motor cortex, we noted that some micro-arousals occurred alongside a brief decrease in firing rate across channels, while other micro-arousals were associated with an increase in multi-unit activity in a similar manner to transitions from sleep to wakefulness (Fig. 2A). Analysis of the durations of automatically-detected micro-arousals across mice showed that most micro-arousals lasted 2–4 s while 30% of micro-arousals were between 5 and 10 s (Fig. 2B). We therefore applied this threshold to all further analysis, where only EMG durations below 10 s were included. We hypothesized that short micro-arousals were associated with a decrease in firing while longer micro-arousals would have more similarity to wakefulness that generally have higher firing rates than NREM sleep^33^. To test this hypothesis, we divided micro-arousals into groups based on their duration so one group consisted of micro-arousals with a duration below 5 s and the other group consisted of micro-arousals with a duration from 5 to 10 s. Comparison of the average firing rate, measured as number of spikes per second from 16 channels located across the cortical column, showed that micro-arousals shorter than 5 s on average were associated with a decrease in firing, while both micro-arousals with durations between 5 and 10 s and transitions from sleep to wakefulness (defined as muscle activity for more than 20 s) exhibited increased firing rates (Fig. 2C, D). Interestingly, both short and longer micro-arousals exhibited a short period of briefly increased activity immediately prior to the beginning of the micro-arousal. This “shoulder” of increased firing before the muscle activity was barely visible during micro-arousals shorter than 5 s but was pronounced prior to longer micro-arousals. Visual inspection of the firing rate within each channel revealed that this dual firing pattern was due to some channels exhibiting an increase in firing immediately prior to the arousal while other channels exhibited a decrease immediately after the beginning of the muscle activity (Fig. S1). Channels with decreased and increased firing during micro-arousals were scattered across the cortical column and did not show any apparent groupings across layers or cortical depths.

To test if the different firing pattern during short and longer micro-arousals was due to increased firing within micro-arousal-active channels or a switch in micro-arousal-inactive channels from silent to active, we collected channels where micro-arousals with a duration below 5 s were associated with either an increase or decrease larger than 2 times the standard deviation. A total of 12 channels from 6 different mice were found to show increased activity during short micro-arousals. These channels did not exhibit a significant difference in firing rate during short compared to longer micro-arousals (Fig. 2E). On the contrary, channels exhibiting decreased firing during short micro-arousals (a total of 89 channels across all seven mice) had significantly higher firing during longer micro-arousals (Fig. 2F). Because the resolution of the multi-unit activity probes does not allow for precise identification of single neurons, it is not possible to say whether the increased firing is the result of a shift in activity of specific neurons from silent to active, or whether it reflects that longer lasting micro-arousals engage a larger number of neurons in proximity to the probe. However, these results indicate that micro-arousals during NREM sleep are not uniform and that very short micro-arousals with a duration below 5 s are more similar to periods of neuronal silence while longer micro-arousals share more similarities with transitions to wakefulness.

### Sleep deprivation is associated with increased cortical firing prior to micro-arousals

**Fig. 3:**
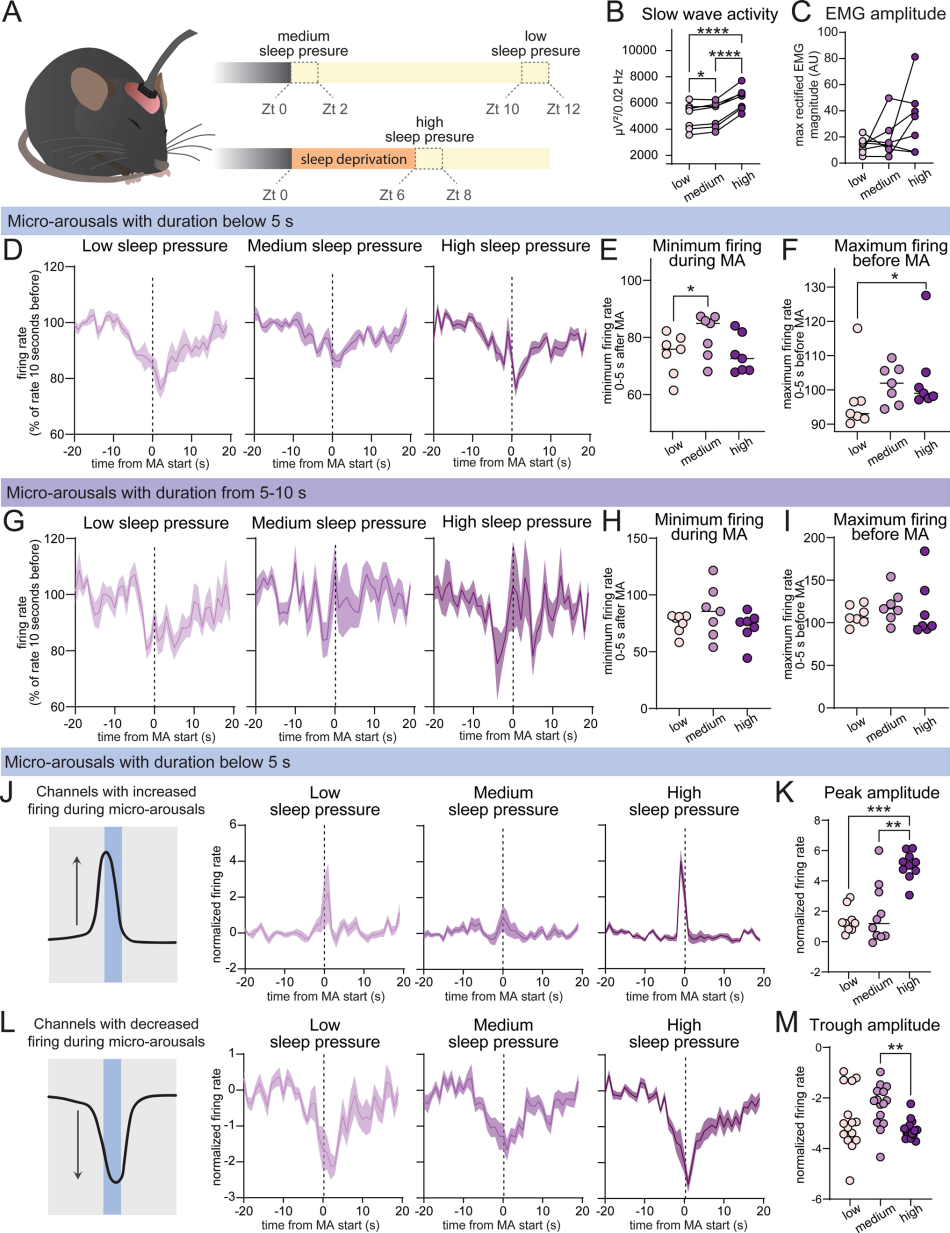
Sleep deprivation is associated with increased cortical firing prior to micro-arousals. (**A**) Diagram showing the three sleep pressure groups. The low and medium sleep pressure groups consist of micro-arousals occurring during the last 2 h and first to hours of the 12 h light period, respectively. The high sleep pressure group consists of micro-arousals occurring during the first two hours following 6 h of sleep deprivation. (**B–C**) NREM SWA (**B**) and amplitude of EMG activity during micro-arousals (**C**) within the low, medium, and high sleep pressure time bin (n = 7, *P* < 0.0001 for low vs. high and medium vs. high, *P* = 0.039 for low vs. medium, RM one-way ANOVA with Geisser-Greenhouse correction and Tukey’s multiple comparison). (**D**) Firing rate during micro-arousals with durations below 5 s, while mice were subjected to low, medium and high sleep pressure (n = 7). (**E**–**F**) Quantification of minimum firing rates 0–5 s after (**E**), and maximum firing rate 0–5 s before (**F**) the beginning of micro-arousals with durations below 5 s (n = 7, P = 0.047 in (**E**) and *P* = 0.017 in (**F**), RM one-way ANOVA with Geisser-Greenhouse correction and Tukey’s multiple comparison). (**G**) Firing rate during micro-arousals with durations between 5 and 10 s while mice were subjected to low, medium and high sleep pressure (n = 7). (**H**–**I**) Quantification of minimum firing rates 0–5 s after (**H**), and maximum firing rate 0–5 s before (**I**) the beginning of micro-arousals with durations between 5 and 10 s (n = 7, RM one-way ANOVA with Geisser-Greenhouse correction and Tukey’s multiple comparison). (**J**) Average firing rate under different sleep pressure conditions for channels exhibiting increased firing during the high sleep pressure condition (total of 10 channels from 4 mice, values are normalized to the standard deviation). (**K**) Amplitude of the peak in firing rates from 5 s before to 5 s after the beginning of micro-arousals arousals (n = 7, *P* = 0.0001 for low vs. high and *P* = 0.002 for medium vs. high, RM one-way ANOVA with Geisser-Greenhouse correction and Tukey’s multiple comparison). (**L**) Average firing rates under increasing sleep pressure for channels exhibiting decreased firing during the high sleep pressure condition (total of 15 channels from 6 mice, values are normalized to the standard deviation). (**M**) Amplitude of the minimum firing rate from 5 s before to 5 s after the beginning of micro-arousals arousals (n = 7, *P* = 0.002 for medium vs. high, RM one-way ANOVA with Geisser-Greenhouse correction and Tukey’s multiple comparison). Shaded area around line graphs are ± SEM, MA = micro-arousal.

Sleep homeostasis refers to the ability to keep track of time spent in sleep and wakefulness, and ensures that the need for an adequate amount of sleep is satisfied. High sleep drive manifests as increased SWA in NREM sleep and an increased threshold to be woken up by external stimuli^25,34–36^. We next set out to investigate if sleep homeostasis affects the neuronal characteristics of micro-arousals by analysing neuronal firing patterns during micro-arousals under conditions of high or low sleep pressure (Fig. 3A). Micro-arousals were pooled into three groups; (1) low sleep pressure (micro-arousals occurring during the last 2 h of the light phase), (2) medium sleep pressure (micro-arousals occurring during the first 2 h of the light phase) and (3) high sleep pressure (micro-arousals occurring during the first 2 h of recovery sleep after 6 h of sleep deprivation by novel objects exposure started at the beginning of the light phase). Neuronal activity around micro-arousals was then compared between the groups. Spectral analysis of layer 5 motor cortex LFP signals confirmed that higher sleep pressure was associated with increased levels of NREM SWA (Fig. 3B). The amplitude of the EMG signal during micro-arousals exhibited a larger variance in the high sleep pressure group, but no significant difference was observed between groups (Fig. 3C).

We first analysed the firing rates during micro-arousals across the different sleep pressure groups for short (< 5 s) and longer (5–10 s) micro-arousals. Comparison of the average firing rate, measured as number of spikes per second from 16 channels spaced across the cortical column, showed that micro-arousals shorter than 5 s were associated with a decrease in firing, which was independent of sleep pressure although the medium sleep pressure group showed a slightly smaller decrease compared to the low sleep pressure group (Fig. 3D, E). However, the “shoulder” of increased firing immediately before the micro-arousal was present in the high sleep pressure group, but not the low sleep pressure group (Fig. 3D, F). On the contrary, longer micro-arousals with durations between 5–10 s exhibited a more variable firing pattern and no significant difference in firing before or after the arousal across sleep pressure groups (Fig. 3G–I). Thus, the analysis indicates that short micro-arousals are modulated by sleep pressure to a higher degree than longer micro-arousals.

To explore how sleep pressure modulates the cortical firing patterns of short micro-arousals, we collected channels exhibiting either increased or decreased firing rate in association with micro-arousals during the high sleep pressure condition. A total of 10 channels from 4 different mice were found to show increased activity. The micro-arousal associated firing rate from these channels did not differ between low and medium sleep pressure conditions, but was significantly increased under high sleep pressure (Fig. 3j, K) with a peak 0.7 ± 0.46 s before the beginning of the micro-arousal. A total of 15 channels from 6 different mice were found to show decreased firing during micro-arousals under the low sleep pressure condition. These channels also exhibited sensitivity to sleep pressure, although the smallest decrease in firing rates were found in the medium sleep pressure group and the most pronounced drop was evident under high sleep pressure (Fig. 3L, M) with lowest values 1.5 ± 2.62 s after the initiation of the micro-arousal.

In summary, analysis of neuronal firing during micro-arousals show that short (< 5 s) micro-arousals are modulated by sleep pressure and that a population of neurons spaced across the motor cortex increase their firing immediately before micro-arousals while another population decrease their activity during micro-arousals. Both populations are modulated by sleep pressure although only the population with increased activity prior to micro-arousals exhibited significantly higher firing under high sleep pressure compared to both the low and medium sleep pressure group. On the contrary, longer micro-arousals exhibited a more variable firing pattern without significant differences across the sleep pressure conditions.

### Post micro-arousal SWA exceeds NREM sleep SWA in sleep deprived mice

**Fig. 4:**
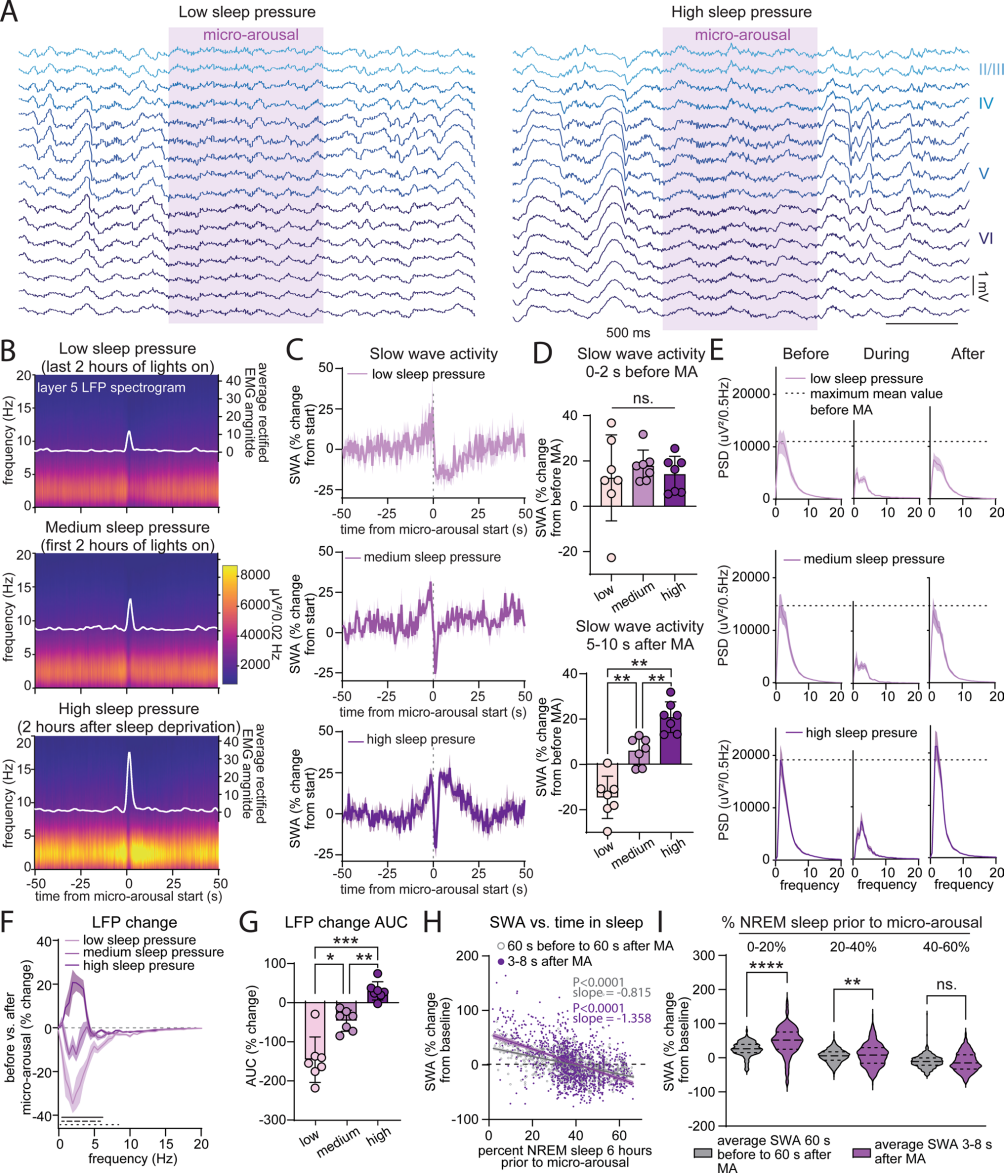
Post micro-arousal SWA exceeds NREM sleep SWA in sleep deprived mice. (**A**) Example motor cortex LFP traces during a micro-arousal under baseline conditions (low sleep pressure, left) and after 6 h of sleep deprivation (high sleep pressure, right). (**B**) Mean LFP spectrograms from layer 5 neurons during low, medium, and high sleep pressure where t = 0 depicts start of EMG activity (n = 7, only micro-arousals with duration below 5 s included). (**C**) SWA expressed as percent change from 20 to 50 s before the micro-arousal during low (top panel), medium (middle panel) or high (bottom panel) sleep pressure (n = 7). (**D**) Average SWA 0–2 s before the micro-arousal (top) and 5–10 s after the micro-arousal (bottom) (n = 7, RM two-way ANOVA with Geisser-Greenhouse correction and Tukey’s multiple comparison, *P* values for SWA after MA: *P* = 0.006 for low vs. medium, *P* = 0.001 for low vs. high, *P* = 0.007 for medium vs. high, error bars are SD). (**E**) Mean power spectral density plots before (t = − 50 to − 30 s), during (t = 0 to 2 s) and after (t = 5 to 10 s) micro-arousals occurring under low (top panel), medium (middle panel) or high (bottom panel) sleep pressure (n = 7, dashed horizontal line shows the maximum value before the micro-arousal). (**F**) Percentage change in power spectral densities before vs. after micro-arousals during low, medium and high sleep pressure. Black line denotes significant difference between medium and high sleep pressure (1–6.5 Hz), black dashed line denotes significant different between low and medium sleep pressure (1–6 Hz) and black dotted line denotes significant difference between low and high sleep pressure (0.5–8.5 Hz) (n = 7, RM two-way ANOVA and Tukey’s multiple comparison). (**G**) Quantification of mean area under the curve for data in (F) (n = 7, *P* = 0.024 for low vs. medium, *P* = 0.0001 for low vs. high, *P* = 0.004 for medium vs. high, RM one-way ANOVA with Geisser-Greenhouse correction and Tukey’s multiple comparison, error bars are SD). (**H**) Percent time spent in NREM sleep during a period 6 h prior to each micro-arousal correlated with the amplitude of SWA around (1 min before to 1 min after, shown in grey) or after (3–8 s after, shown in purple) the same micro-arousal. SWA amplitude is shown as percent of mean NREM SWA during the first 6 h of lights on. 1162 micro-arousals from 7 mice during 18 h after 6 h sleep deprivation included. The lines show the linear regression and the shaded area depict 95% confidence intervals. Comparison of the slopes with linear regression showed a significant difference (Pearson correlation, *P* < 0.0001, ANCOVA). (**I**) Comparison of average SWA (shown as the percent change from NREM SWA during the first 6 h of the light phase on the baseline day) during a period 1 min before to 1 min after each micro-arousal and during a period 3–8 s after each micro-arousal. The micro-arousals are divided into bins after the amount of NREM sleep that was present 6 h before the micro-arousal (n = 7 with 116 micro-arousals in the 0–20% bin, 529 micro-arousals in the 20–40% bin, and 457 micro-arousals in the 40–60% bin, Paired *t* test, *P* < 0.0001 for 0–2% and *P* = 0.001 for 20–40%). Shaded area around line graphs are ± SEM. MA = micro-arousal, SWA = slow wave activity, LFP = local field potential.

Are the neuronal oscillatory patterns during micro-arousals affected by sleep pressure? To answer this, we analysed local field potential (LFP) traces within the motor cortex. All cortical layers analysed (layer 2/3, 4, 5, and 6) displayed a ~ 50–70% drop in LFP power during micro-arousals with the most pronounced drop happening in the slow wave activity (SWA) / delta (1–4 Hz) frequency range (Fig. S2A-D). Although this was evident within all layers, layer 5 exhibited a slightly larger drop compared to layer 2/3 (Fig. 4S2E). To investigate the effect of sleep pressure on the LFP signals, we analysed LFP power in layer 5 around micro-arousals within the same three groups of increasing sleep pressure; (1) low sleep pressure (micro-arousals occurring during the last 2 h of the light phase), (2) medium sleep pressure (micro-arousals occurring during the first 2 h of the light phase) and (3) high sleep pressure (micro-arousals occurring during the first 2 h of recovery sleep after 6 h of sleep deprivation by exposure to novel object started at the beginning of the light phase) (Fig. 4A). Because we saw a link between sleep pressure and short (< 5 s duration) micro-arousals, only micro-arousals with a duration below 5 s were included in this analysis. Analysis of LFP power revealed that micro-arousals exhibited an increase in SWA right before the micro-arousal, which did not differ between the sleep pressure group (Fig. 4B–D). However, SWA immediately after the micro-arousal was highly dependent on sleep pressure; the low sleep pressure group exhibited a long period with decreased SWA following micro-arousals, while the high sleep pressure group exhibited a pronounced SWA increase with SWA levels exceeding those before the micro-arousal (Fig. 4D). SWA after micro-arousals from the medium sleep pressure group was in between the SWA levels of the groups of low and high sleep pressure. Power spectral density analysis of the period before (30–50 s before muscle activity), during (0–2 s after the first muscle activity), and after (5–10 s after the first muscle activity) each micro-arousal showed that all groups exhibited a decrease in power during the micro-arousal (Fig. 4E). In addition, the analysis confirmed that the low and medium sleep pressure groups showed reduced SWA levels after micro-arousals, while the high sleep pressure group showed higher SWA levels after the arousal than before. The percentage change in power spectral densities before versus after the micro-arousal was highly sensitive to sleep/wake history (Fig. 4F). Both the low and medium sleep pressure group exhibited a decrease in low frequencies after compared to before the micro-arousal. The low sleep pressure group displayed significantly larger negative changes than the medium sleep pressure group in frequencies between 1 and 6 Hz. Contrary to the low and middle sleep pressure group, the high sleep pressure group displayed a significant positive change in power spectral densities from before to after the micro-arousal. The difference between low and high sleep pressure spanned a frequency range from 0.5 to 8.5 Hz. This sleep-pressure sensitive shift was also evident when the percentage change was plotted as the area under the curve (Fig. 4G). Analysis of the duration of micro-arousals during a 24-h baseline recording compared with a period 2 h after sleep deprivation showed that this was not due to differences in the duration of micro-arousal between low and high sleep pressure conditions (Fig. S3A). The frequency of < 5 s long micro-arousals showed a trend towards increasing over the time course of the recovery sleep following sleep deprivation, although this did not reach statistical significance (Fig. S3B).

It is well-established that NREM sleep SWA reflects time spent awake in the period prior to sleep^25,26,35^. We next asked if the period immediately following micro-arousals follow the same dynamics. First, all micro-arousals during a period of 18 h following 6 h sleep deprivation with novel objects (1162 micro-arousals from 7 mice) were collected for analysis. The level of SWA 3–8 s after each micro-arousal was then plotted against the percent time spent in NREM sleep during 6-h prior to the micro-arousal (Fig. 4H). Post-arousal SWA exhibited a significant negative correlation with prior time spent in sleep, meaning that less sleep resulted in higher SWA, in accordance with the established link between SWA and sleep homeostasis. To investigate the relationship between SWA during a longer period of NREM sleep compared to post-arousal SWA, we calculated SWA during a 2-min period around each micro-arousal (1 min before to 1 min after) and performed the same correlation with time spent in sleep within 6 h prior to the arousal. Post-arousal SWA (3–8 s after the micro-arousal) exhibited a significant positive correlation with SWA during the 2-min period around the micro-arousal (Fig. S3C). However, the slope of the correlation between sleep time and post-arousal SWA was significantly steeper than the slope of the correlation between sleep time and SWA 1 min before to 1 min after the micro-arousal (Fig. 4H). This indicates that post-arousal SWA is more sensitive to homeostatic sleep pressure than general NREM sleep SWA. We found that post-arousal SWA was significantly higher than SWA 1 min before to 1 min after the micro-arousal when the mice had only spent 0–20% and 20–40% of the past 6 h in sleep but not if they had spent 40–60% in sleep (Fig. 4I). In addition, this effect was stronger for mice in the 0–20% group than the 20–40% group, indicating that the difference between post-arousal SWA and NREM SWA is more pronounced with increasing levels of sleep pressure. In other words, well-rested mice exhibited similar levels of SWA after micro-arousals and during NREM sleep, whereas sleep deprived mice exhibited higher SWA levels after micro-arousals than the average SWA level during recovery sleep.

To investigate if micro-arousals are associated with the sleep rebound after sleep deprivation, we calculated the decrease in NREM SWA within the first two hours of recovery sleep after sleep deprivation as an estimate of how efficiently SWA is restored back to a baseline level. The decrease in SWA following sleep deprivation significantly correlated with the amount of time spent in NREM sleep but not with the frequency of micro-arousals (Fig. S3D-E). This indicates that micro-arousals are not directly associated with recovery from sleep deprivation. We also performed the LFP analysis on longer micro-arousals with durations between 5–10 s but did not find the same close association between post-arousal SWA and sleep pressure although we did find a significant difference in SWA before the micro-arousal between the low and medium sleep pressure group and a significant difference in post-arousal SWA between the medium and high sleep pressure group (Fig. S3F-G). Finally, we tested whether the sleep pressure-associated increase in post-arousal SWA was specific to certain cortical layers by comparing SWA during micro-arousals in the low and high sleep pressure group for layer 2/3, 4, 5, and 6 (Fig. S3H). All layers exhibited a pronounced increase in SWA from the low to high sleep pressure condition. Calculation of the increase in post-arousal SWA from the low to high sleep pressure condition showed an overall significant difference between the layers (*P* = 0.045) and layer 5 exhibited a significantly higher SWA increase compared to layer 2/3 and layer 4 (Fig. S3H) in the post-hoc test. Overall, these results show that micro-arousals are preceded by an increase in SWA that is not affected by sleep pressure but that post-arousal SWA is modulated by sleep homeostasis and exceeds SWA levels of sustained NREM sleep in sleep deprived mice.

### Neuronal activity during micro-arousals following REM sleep differs from NREM sleep micro-arousals

**Fig. 5:**
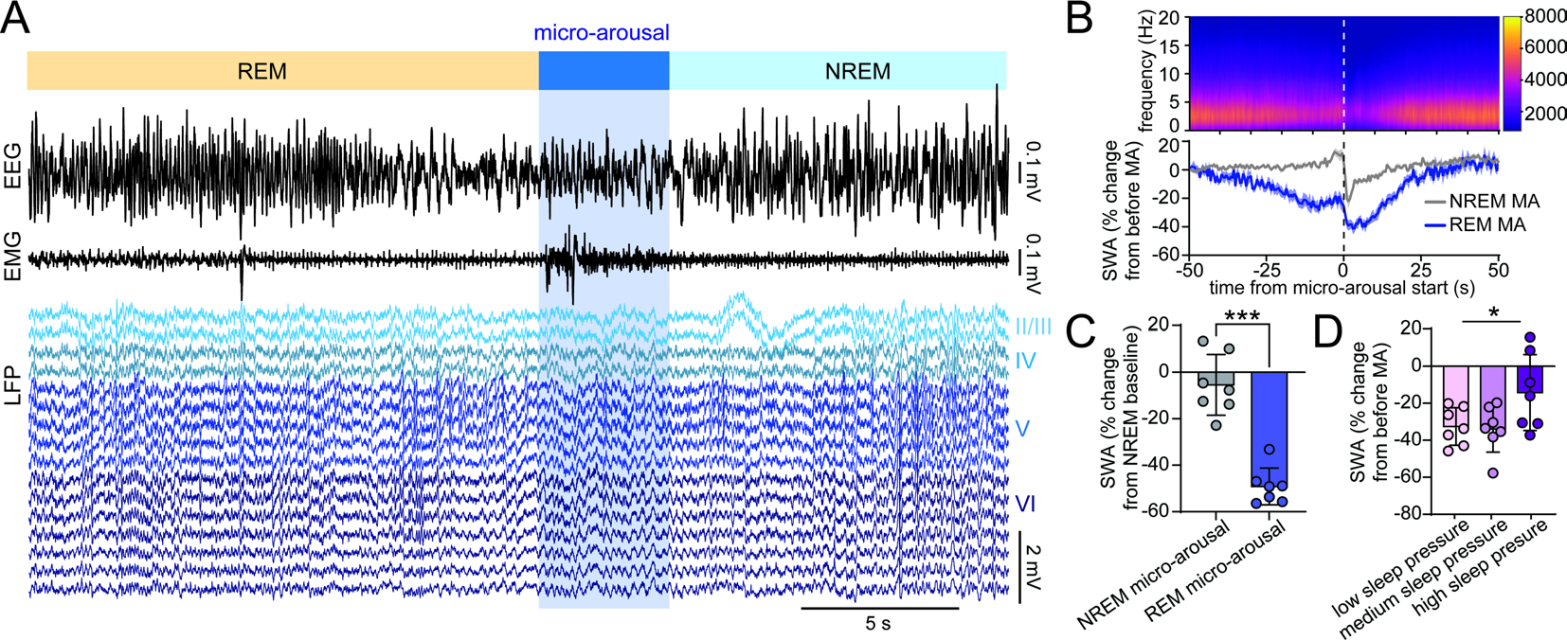
Neuronal activity during micro-arousals following REM sleep differs from NREM sleep micro-arousals. (**A**) Example of EEG, EMG, and motor cortex LFP traces showing REM sleep termination followed by a micro-arousal and transition to NREM sleep. (**B**) LFP spectrogram (top) and mean SWA expressed as percent change from 50 to 20 s before the micro-arousal (bottom) with t = 0 depicting the beginning of the micro-arousal (n = 7, shaded area is ± SEM). (**C**) Mean SWA 0–5 s after onset of NREM sleep- and REM sleep micro-arousals as percent of baseline NREM SWA (n = 7, *P* = 0.0003, Paired *t* test, all micro-arousals during the 24 h baseline recording included, error bars are SD). (**D**) Mean change in SWA from 50 to 20 s before to 5–10 s after REM sleep-terminating micro-arousals during low, medium and high sleep pressure. To include enough micro-arousals, the low sleep pressure group consists of micro-arousals during the last 6 h of the baseline light period and the medium sleep pressure group consists of micro-arousals during the first 6 h of the baseline light period (n = 7, RM one-way ANOVA with Geisser-Greenhouse correction and Tukey’s multiple comparison, Repeated measures effect *P* = 0.0435, no significant effect in the multiple comparisons test, error bars are SD). MA = micro-arousal, SWA = slow wave activity, LFP = local field potential.

Micro-arousals occur regularly during NREM sleep but are also commonly observed after bouts of REM sleep upon the transition back to NREM sleep. Evidence suggests that micro-arousals during NREM sleep and micro-arousals at the termination of REM sleep are both associated with an increase in locus coeruleus activity and a subsequent increase in brain noradrenaline levels^13^. However, how similar the two types of micro-arousals are in terms of neuronal dynamics and sensitivity to sleep homeostasis is not well established. We used an altered version of the micro-arousal detection algorithm to only detect micro-arousals that followed REM sleep and analysed the neuronal activity patterns. Slow waves were regularly observed in the layers of the motor cortex during REM sleep, as previously described^37,38^ (Fig. 5A). Spectral analysis of micro-arousals at the termination of REM sleep showed a gradual decrease in SWA before the micro-arousal, which was followed by a gradual increase in SWA after the transition to NREM sleep (Fig. 5B). REM sleep micro-arousals exhibited significantly lower SWA levels than NREM sleep micro-arousals (Fig. 5C). Finally, there was an overall significant difference between post-micro-arousal (5–10 s after micro-arousal initiation) SWA under different sleep pressure conditions (first 6 h of lights on, last 6 h of lights on (6 h time windows were used here to include enough bouts of REM sleep), and first 2 h after 6 h sleep deprivation) but no significant difference between the individual sleep pressure conditions in post-hoc analysis (Fig. 5D).

## Discussion

We here presented an algorithm to automatically detect sleep micro-arousals based on EMG activity, and used it on data from mice implanted with EMG and EEG electrodes as well as an LFP/multi-unit activity probe placed in the motor cortex to study cortical firing dynamics during micro-arousals. Analysis of single channel firing showed a non-uniform firing pattern where short (< 5 s duration) micro-arousals were associated with a decrease in firing while longer micro-arousals (5–10 s) exhibited an increase in firing in a similar manner to transitions from sleep to wakefulness (Fig. 2). We found that some channels exhibited increased activity immediately prior to the micro-arousal while other channels showed decreased firing during micro-arousals. The increased firing immediately before the arousal was present when the mice were under high sleep pressure, but not when they were well rested (Fig. 3). Interestingly, LFP analysis showed that short micro-arousals were preceded by an increase in SWA irrespectively of the levels of sleep pressure (Fig. 4). On the contrary, SWA immediately after the micro-arousal was highly sensitive to sleep/wake history, such that mice experiencing low sleep pressure exhibited a long (~ 20 s) period of decreased SWA after micro-arousals whereas mice under high sleep pressure exhibited an overshoot in SWA immediately following the arousals. This change could not be explained by a difference in the duration of micro-arousals between the conditions and was evident in all cortical layers analysed (layer 2/3, 4, 5, and 6) but with the largest effect within layer 5. Importantly, the rate by which SWA decreased as a function of time spent in sleep was steeper for SWA immediately after micro-arousals than for SWA within a period of NREM sleep from 1 min before to 1 min after the micro-arousal. Finally, we found that micro-arousals after REM sleep differed from NREM sleep micro-arousals in terms of neuronal activity patterns and relationship with sleep homeostasis (Fig. 5).

Our analysis showing that short bouts of muscle activity share more similarities with neuronal activity during NREM sleep, which is characterised by frequent occurrences of population OFF periods^33^, while longer bouts of muscle activity share more similarities with a transition to wakefulness in terms of neuronal firing, indicates that the heterogeneity of micro-arousals may be larger than what is currently established. Furthermore, it underscores the notion that micro-arousals per se are not equivalent to short periods of wakefulness. This is supported by another study where recordings of neuronal firing in the frontal cortex of rats showed that micro-arousals increased the firing rate of slow-spiking neurons during the subsequent NREM sleep epoch, while epochs of wakefulness had the opposite effect^39^. Interestingly, other studies have previously reported the existence of different subtypes of arousals with different properties, although these distinctions have mainly been made based on the presence or absence of muscle activity during the arousal. Thus, a study in humans combining EEG recordings, PET scans for assessment of amyloid-β burden, and cognitive tests found that cortical arousals (defined as a shift in EEG frequencies for more than 3 s) associated with a sleep stage transition and no muscle activity was positively correlated with brain amyloid burden. On the contrary, the frequency of cortical arousals with concurrent muscle activity was associated with lower amyloid burden and higher cognitive performance^40^. Studies in mice have shown that NA release during NREM sleep not always results in movement and wake-like EEG, but sometimes instead elicits an increase in slow frequencies^13,16^. In our study, micro-arousals were detected solely based on muscle activity and we did therefore not include analysis of arousals without muscle activation. However, it is possible that the very short duration of muscle activity during the short (5 < s duration) micro-arousals, which were sometimes less than a second long, in some studies not have been regarded as actual muscle activity. Thus, the 5 < s micro-arousals investigated in this study may have similar properties to the cortical arousals described in some previous studies.

The analysis of single-channel activity during micro-arousals revealed that some channels exhibited increased activity immediately prior to the first muscle activity, while other channels decreased their activity. A proper identification of single neuronal subtypes would require higher spatial resolution than the 16 channel probes used in this study but could be obtained in future studies using higher density probes. Especially the group of neurons exhibiting increased activity before the arousal would be interesting to identify, as these may have a role in the initiation of arousals. Thus, the identification and exploration of these neurons in health and disease states may be able to give insights as to why old age and certain states of pathology are associated with an increased arousal burden.

Our analysis identified a strong correlation between sleep/wake history and SWA in the period immediately after short NREM sleep micro-arousals. Surprisingly, the slope of this correlation was steeper than the correlation between sleep/wake history and SWA during a longer period of NREM sleep before and after the micro-arousal, indicating that post-arousal SWA is more sensitive to sleep/wake history than SWA during more sustained NREM sleep. The homeostatic increase in NREM SWA in response to high sleep pressure is well-known although its functional significance is still not clear^24–26^. Given our observation that sleep deprived mice exhibited higher SWA levels in association with micro-arousals than during regular NREM sleep, it could be speculated whether micro-arousals play a role in recovery sleep after prolonged wakefulness. However, our analysis showed that the rate of SWA decrease during rebound sleep after sleep deprivation was correlated with time spent in NREM sleep but not the frequency of micro-arousals. Another potential interpretation of the finding that post-arousal SWA was higher in sleep deprived mice could be that it reflects a mechanism that works to terminate the arousal in order to resume sleep. This would explain the increase in SWA as a function of prior time spent in wakefulness, as higher sleep need would impose higher sleep pressure and thereby higher incentive to return to sleep. However, this mechanism would be expected to lead to shorter micro-arousals during high sleep pressure conditions, which we did not observe here. With the tight link between NA and micro-arousals, it is possible that changes in NA dynamics play a role in the increased SWA observed after sleep deprivation. Indeed, NA levels have been shown to decline gradually during sleep and increase during wakefulness^41^, while the amplitude of the NA oscillation is higher after sleep deprivation than during baseline sleep^16,42^. Future studies using combined recordings of NA and neuronal activity after sleep deprivation could provide further insights to this question.

It is also worth noting that while increased SWA after micro-arousals only was observed in sleep deprived mice, all groups exhibited an increase in SWA immediately prior to micro-arousals. What mainly separates the pre-arousal and post-arousal period is that they are on different sites of the rise in NA that promotes the micro-arousal^13^. In other words, the brain has the lowest NA levels during the pre-arousal period and the highest NA levels during the post-arousal period. Evidently, this means that neuronal slow waves before the micro-arousal will happen under very low levels of NA, while slow waves after the micro-arousal will happen while NA release is at its maximum. This difference could impact the downstream processes associated with SWA, such as memory formation. Interestingly, a recent study in humans found that the arousal cycle organizes slow waves in two distinct clusters; a cluster with high amplitude and high degree of synchronization across the scalp, which primarily occurs during high NA levels, and a cluster with smaller and less synchronized slow waves that were more evident during low-arousal periods^43^. NA has been shown to promote long term potentiation (LTP) in the hippocampus^44–46^ and long term depression (LTD) in the dentate gyrus^47^, and reducing the overall levels of NA specifically during sleep leads to impaired memory performance, while enhancing NA during sleep promotes memory formation^48^. Thus, the tightly regulated variability in NA in addition to the hypersynchronous slow firing across the cortical column before and after micro-arousals may provide a window for cognitive processing. This notion is supported by the finding that increased cross-talk between the cortex and the hippocampus takes place during the period leading up to micro-arousals^17^.

To our knowledge, this is the first study to identify a relationship between neuronal activity during micro-arousals and sleep/wake history. The frequency of arousals from sleep has previously been shown to be partly under homeostatic control. Thus, one study reported that 64 h sleep deprivation in healthy adults resulted in a significant decrease in micro-arousals, here defined as a shift to faster EEG frequencies within a period of 1.5–3 s, and a trend towards a decrease in cortical arousals associated with K-complexes or increased delta waves^27^. On the contrary, arousals associated with muscle activation were unaltered by sleep deprivation. A study in rats reported that 12 h of sleep deprivation led to a reduction in the total number of brief awakenings (defined as wakefulness for up to 16 s) during subsequent recovery sleep and found a significant negative correlation between SWA and the frequency of awakenings^9^. A study in mice tested the effect of 4 or 6 h sleep deprivation on the frequency of brief awakenings (defined as wakefulness for up to 16 s) but only found a robust decrease in brief awakenings after 6 h of sleep deprivation, while the number of brief awakenings were not significantly affected by 4 h of sleep deprivation in two of the three mouse strains tested^29^. In addition, this study only found a negative correlation between SWA and the number of brief awakenings in one out of the three mouse strains. Another study reported that sleep deprivation, using methods that are stressful for the mouse in this case cage shaking, increased both SWA and the number of micro-arousals (defined as muscle activity and faster EEG frequencies for < 12 s) during subsequent sleep^16^. Thus, the relationship between sleep homeostasis and micro-arousal generation seems to not be straight forward and depend on more factors than the time spent in wakefulness prior to sleep. An interesting next step will be to investigate how the sleep pressure-associated changes in neuronal activity during micro-arousals is affected by experiences during prior wakefulness and to untangle why the number of micro-arousals is negatively correlated with SWA in some studies and not in others. Our analysis of the frequency of micro-arousals during recovery sleep after sleep deprivation showed a trend toward fewer micro-arousals in the beginning of the sleep period compared to the end, although this difference was not significant (Fig S3B). However, the algorithm used for the detection of micro-arousals in this study was not developed to precisely analyse micro-arousal quantity, but rather to detect micro-arousals in order to study the associated cortical activity pattern. For example, micro-arousals were omitted from the analysis if they were within 20 s of another micro-arousal, in order to ensure that the analysed micro-arousals were surrounded by stable NREM sleep. This may explain why we did not observe the same relationship between sleep pressure and micro-arousal frequency as reported in other studies.

Our study suggests that micro-arousals are a heterogeneous phenomenon, and that their properties depend on several factors, including sleep stage, sleep/wake history and duration. For example, REM sleep micro-arousals did not exhibit the increase in SWA prior to the arousal as was seen for NREM arousals, and they did not evoke the same pronounced increase in SWA upon transition back to NREM sleep under high sleep pressure conditions. In addition, short micro-arousals (< 5 s) were associated with an overall decrease in cortical neuronal firing, while this was not the case for longer micro-arousals (5–10 s). This suggests that arousals of longer duration is more similar to sustained wakefulness, where firing rates are generally higher^33^, while very short bouts of muscle activity exhibited more similarities with neuronal activity during NREM sleep, which is characterised by frequent occurrences of population OFF periods^33^.

There is currently a significant translational gap between the study of micro-arousals in rodents and humans. One of the reasons for this is the discrepancy between how arousals are traditionally defined across human and rodent studies. Thus, scoring of arousal events in humans is mostly focused on changes in EEG frequencies^49^, while rodent arousal scoring more often is focused on the associated motor response. Additionally, scoring criteria for arousals in rodents is not standardized and therefore often differs between studies, which makes direct comparison of results from different studies difficult. The idea that sleep arousals exist as a range of physiological responses that most likely arise from the same mechanism is not new, and the use of fluorescent biosensors allowing for the detection of neurotransmitter release on the millisecond scale in mice has confirmed this notion. However, the “how” and “why” of micro-arousals are still not resolved, and until they are, it will be difficult to establish a stern definition of them.

Another reason for why the study of micro-arousals is still at a premature stage is the common misconception, found in both human and rodent studies, that micro-arousals by default are a sign of poor and fragmented sleep. This view is supported by studies showing that acoustically induced arousals during sleep lead to increased sleepiness and reduced performance during the following wake period^50,51^. However, most micro-arousals are internally-generated and occur in the absence of external stimuli^1^. Therefore, externally and internally-initiated arousals most likely do not have the same implications for sleep continuity and quality. This is supported by a recent study that showed different anatomical activation pathways for sensory-evoked awakenings and spontaneous awakenings^52^. On the other hand, sleep disorders, such as sleep apnoea and restless leg syndrome, where repeated awakenings have detrimental effects on the wellbeing of the patient, exhibit a clear cyclical pattern in the generation of arousals, indicating that the infraslow arousal pattern contribute to the disease phenotype^53,54^. Identification of traits that distinguish micro-arousals during healthy sleep and arousals associated with pathological states will be important to detect early signs of pathology, which often manifests as changes in sleep before other clinical signs become evident.

In conclusion, we show that short, EMG-defined micro-arousals are associated with a decrease in cortical firing, while longer micro-arousals occur alongside an increase in firing. The cortical activity pattern associated with micro-arousals is non-heterogeneous with some channels exhibiting increased firing prior to micro-arousals while other channels exhibit decreased firing during micro-arousals. Furthermore, analysis of LFP power throughout the layers of the motor cortex showed that increased SWA in response to sleep pressure is more pronounced immediately after the micro-arousal than during NREM sleep but that this is not the case for micro-arousals following REM sleep. The combined set of data shows that micro-arousals are heterogeneous in their appearance and are intimately related to homeostatic regulation of sleep. This finding provides a basis for identifying physiological differences between micro-arousals in healthy, restorative sleep and arousals with detrimental effects on sleep quality and function.

## Methods

All experiments were performed in accordance with the United Kingdom Animal Scientific Procedures Act 1986 under personal and project licenses granted by the United Kingdom Home Office and the ARRIVE guidelines. Ethical approval was provided by the Ethical Review Panel at the University of Oxford. Animal holding and experimentation were located at the Biomedical Sciences Building and the Behavioural Neuroscience Unit, University of Oxford. Chronic electrophysiological recordings from 7 wild-type C57BL/6 mice (internal breeding colony, Biomedical Services, University of Oxford, 125 ± 8 d old at the time of recording) were analysed. This dataset was used in three previous publications^30–32^. In brief, mice were surgically implanted with EEG electrodes for recordings of frontal and occipital regions and a 16-channel silicone probe (NeuroNexus Technologies; A1 × 16-3mm-100–703-Z16, 100 µm spacing between channels) in the motor cortex (+ 1.1 mm AP, − 1.75 mm ML, tilt − 15°). An electrode was implanted in the cerebellum (0.5–1 mm in the midline behind lambda) as reference for the EEG/EMG electrodes and the silicone probe. A pair of stainless-steel wires were inserted into the nuchal muscle for recording EMG. Following the implantation, mice were housed individually with *ad-libitum* access to food and water for a recovery period of around one week. At the end of the experiments, mice were killed using a pentobarbital overdose.

### Data collection and signal pre-processing

Sleep recordings were obtained from custom-made Plexiglas cages, which were placed in sound-attenuated and light-controlled Faraday chambers (Campden Instruments) with a 12:12h light:dark cycle and temperature maintained at around 22 °C. Mice had *ad-libitum* access to food and water throughout the recordings. After a three-day acclimatization period where animals habituated to the recording cage and cable, a 24h baseline recording was started at light onset. On the subsequent day at light onset, mice were subjected to 6 h sleep deprivation by introduction of novel objects to the cage. At the end of the 6 h, all objects were removed from the cage and the mice were allowed to sleep undisturbed.

The electrophysiological data was acquired using a 128-channel Neurophysiology Recording System (Tucker-Davis Technologies) and the electrophysiological recording software Synapse (Tucker-Davis Technologies), and saved on a local computer. EEG and EMG signals were filtered between 0.1 and 100 Hz, and stored at a sampling rate of 305 Hz. Local field potentials (LFPs) for each channel were recorded at a sampling rate of 25 kHz and filtered between 300 Hz and 5 kHz. EEG, EMG and LFP signals were resampled at a sampling rate of 256 Hz using custom-made MATLAB code (MathWorks, v2017a) and converted into European Data Format as previously described^55^.

### Scoring of vigilance state

Vigilance states were scored manually by visual inspection of the EMG and frontal and occipital EEG signals at a resolution of 4 s (SleepSign, v3.3.6.1602, Kissei Comtec). Vigilance states were classified as waking (low amplitude, high frequency EEG with EMG activity present), NREM sleep (high amplitude and low frequency EEG with low EMG activity) or REM sleep (low amplitude, high frequency EEG with increased theta activity in the occipital EEG and low EMG).

### Histology

The tips of laminar implants were stained with DiI (Thermo Fisher Scientific) before surgery by immersion of the electrode shank into a 20 mg ml − 1 solution (50/50% acetone/methanol) for later histological assessment. After completion of the experiments, micro lesions of selected channels on the laminar probe were performed under terminal pentobarbital anaesthesia using the electroplating device NanoZ (White Matter) applying 10 mA of direct current for 25 s to each respective channel. Immediately following micro lesioning, mice were perfused with 0.1 M PBS (0.9%) followed by 4% paraformaldehyde in PBS for tissue preservation. 50-μm thick coronal sections were cut with a vibratome (Leica VT1000S) and brains were stained with DAPI and mounted on glass slides. Fluorescence microscopy was used to map the depth of the laminar probe implantation and the micro-lesions were used to map the location of specific channels within the cortical layers.

### Micro-arousal detection

In order to make the analysis of neuronal activity around micro-arousals as un-biased as possible, the detection of micro-arousals was based solely on the EMG signal and not the EEG signals. The analysis was mainly focused on micro-arousals during NREM sleep, so micro-arousals following REM sleep were not included, except in a version of the algorithm used specifically to detect micro-arousals after REM sleep. Furthermore, the algorithm was developed to detect micro-arousals suitable for analysis of neuronal activity before, during, and after the arousal. Therefore, micro-arousals were only included if a sufficient amount of undisturbed NREM sleep (20 s or more) was present before and after each arousal. A variety of methods are used for vigilance state scoring and range from fully- or semi-automatic scoring (see e.g. ^56–59^) to manual scoring where vigilance states are assigned by visual inspection. Therefore, the goal was not to develop an algorithm for general sleep scoring, but one that could be used on already scored data to extract information about micro-arousals specifically.

As a first step, intruding periods scored as wakefulness or REM sleep during NREM sleep were re-scored as NREM sleep if the duration of the period was below 11 s. This was done to remove existing manually scored micro-arousals. The EMG signal was then filtered and rectified using the moving standard deviation, the moving mean and a 10000th order median filter. Peaks in the EMG signal was detected if the signal crossed a threshold of 2 * the standard deviation. In the subsequent steps, EMG peaks were excluded if they did not occur within periods of NREM sleep, EMG peaks that were less than 10 s apart were combined as one event, and EMG peaks that occurred within 20 s of each other were omitted. Finally, EMG peaks with a duration above 10 s were excluded. As a result, a vector with the beginning of each micro-arousal and a vector with each micro-arousal duration was generated and stored.

The detection algorithm was validated by visual inspection of the EMG and EEG signal and the automatically scored micro-arousals for each mouse, as well as calculation of the average muscle activity during automatically detected micro-arousals compared to micro-arousals scored by manual scoring (Fig. 1).

For detection of micro-arousals following REM sleep, the algorithm was adapted to only include micro-arousals that occurred within 4 s after REM sleep termination. Because EMG activity after REM sleep showed more variability across mice, thresholds for detection of EMG peaks were decided for each mouse after visual inspection of the detected micro-arousals.

### Data analysis

All data analysis was performed in MATLAB (R2024A). Analysis of LFP signals within specific cortical layers were performed on a single channel confirmed to be within the layer of interest from post-mortem histological analysis.

Prior to spectral analysis, data traces were filtered using a 20th order bandpass Butterworth IIR filter with a lower cut-off at 1 Hz and a higher cut-off at 30 Hz. The lower cut-off of 1 Hz, opposed to 0.5–4 Hz which is often used as the cut-off^25,60,61^, was chosen to exclude potential low frequency artefacts caused by movement during micro-arousals.

#### Spectrograms

Spectrograms were generated using the “spectrogram” function with a window of 500 ms and an overlap of 0. Mean power within specific power bands was calculated from the spectrogram by averaging the power within the frequencies of interest. Power spectral density (PSD) plots were calculated using pwelch with a frequency resolution of 0.5 Hz, zero overlap and a Hanning window. PSD plots from before, during and after a micro-arousal were calculated 30–50 s before the micro-arousal, 0–2 s after the beginning of the micro-arousal and 5–10 s after the beginning of the micro-arousal, respectively. EEG frequency bands were calculated by averaging the power of the spectrogram within their respective frequencies (SWA 1–4 Hz, theta 4–10 Hz, sigma 12–16 Hz, beta 16–30 Hz and gamma 30–80 Hz).

#### Correlation between SWA and sleep/wake history

For plotting the correlation between sleep/wake history and post-micro-arousal SWA, all micro-arousals with a duration below 5 s occurring after 6-h sleep deprivation were detected and used in the analysis (sleep deprivation started at lights on so micro-arousals during the 6 last hours of lights on and 12 h during lights off were included). SWA 3–8 s after the beginning of each micro-arousal was first calculated and normalized to the level of SWA during the light phase of the baseline recording from the same mouse. Then, the percent time the mouse had spent in NREM sleep during the 6 h before each micro-arousal was calculated, and the normalized post-micro-arousal SWA was plotted against the percent time spent in NREM sleep. To plot the relationship between SWA during more sustained NREM sleep and sleep/wake history, SWA during a period from 1 min before to 1 min after each micro-arousal was calculated and normalized to SWA during the light phase of the baseline recording. This value was plotted against the percent time the mouse had spent in NREM sleep prior to the micro-arousal.

#### Correlation between SWA decrease rate, NREM amount and micro-arousal frequency

For calculating the decrease in NREM SWA after sleep deprivation, spectrograms were calculated for the first 6 h of recovery sleep after the 6-h sleep deprivation period. The average SWA was then calculated from the spectrogram of the first NREM bout longer than 5 min and the spectrogram of the last NREM bout longer than 5 min. The decrease in SWA from the first to last NREM bout within the 6 h time period was then calculated as the percent change and correlated with the total duration of NREM within the same 6 h or the average number of micro-arousals per hour, respectively.

#### Calculation of average firing rates

The average firing rate from multi-unit recordings was calculated as the combined number of spikes from all channels per second. The firing rates were first calculated for each micro-arousal and then averaged across micro-arousals. For comparison between mice, the averaged firing rate was normalized as the percentage change from the first 10 s of the trace (10 to 20 s before the micro-arousal).

Analysis of single-channels was performed on the averaged firing rates. To compare firing rates across animals and sleep pressure conditions, the firing rate during micro-arousals under the high sleep pressure condition was used to calculate the standard deviation of the firing rate and the mean of the first 10 s for each mouse. These values were subsequently used to normalize the firing rates for all conditions by dividing with the standard deviation and subtracting the mean.

For comparison of single channels across sleep pressure conditions, channels with decreased firing were detected by collecting all channels during the high sleep pressure condition where the minimum value during a period from 5 s before to 5 s after micro-arousals with duration below 5 s was below the mean firing rate 5 to 15 s before the micro-arousal minus 5 times the standard deviation. Likewise, channels with increased firing during the high sleep pressure condition were detected by collecting all channels where the maximum value during a period from 5 s before to 5 s after the micro-arousal was above the mean firing rate 5 to 15 s before the micro-arousal plus 5 times the standard deviation. The firing rate of channels detected as increased or decreased during the high sleep pressure condition were then compared to the firing rate of the same channels during low and medium sleep pressure conditions.

Comparison of single channels between different micro-arousal durations followed the same method, except normalization values were calculated for micro-arousals with a duration below 5 s, and all micro-arousals during the 24-h baseline period were used. Furthermore, the criteria for selection of channels with increased or decreased firing was based on firing above or below 2 times the standard + /- the mean firing rate 5 to 15 s before the micro-arousal was used. This was to ensure a sufficient inclusion of channels as fewer channels showed increased firing during short micro-arousals. Calculation of average firing during transitions to wakefulness was performed as described above, but using an altered version of the micro-arousal detection algorithm where only muscle activity bouts with a duration of more than 20 s was included.

#### Calculation of average rectified EMG

Average rectified EMG traces were calculated by taking the mean of the filtered and rectified EMG trace time-locked to each micro-arousal.

### Statistics

Statistics were done in GraphPad Prism (version 10.3.1). *P* values less than 0.05 (after correction for multiple comparison except the comparison of power density per frequency bins) were considered significant. For comparison of the means of two group where the groups consisted of the same mice under different conditions or time bins, a two tailed paired *t* test were used. For comparison between the means of three or more groups, RM one-way ANOVA with Geisser-Greenhouse correction and Tukey’s multiple comparison was used. For comparison of power densities across frequency bins RM two-way ANOVA with matched values stacked into sub columns was used. Pearson correlation was used to calculate correlation coefficients and slopes of the correlations. ANCOVA was used to test for significant difference between linear regression lines.

## Supplementary Information

Below is the link to the electronic supplementary material.

Supplementary Material 1

## Data Availability

The micro-arousal detection algorithm code is available on GitHub: [https://github.com/NHauglund/automatic-micro-arousal-detection/](https:/github.com/NHauglund/automatic-micro-arousal-detection) . The datasets analysed in the current study is available from the corresponding author on reasonable request.
