# Supplementary material for: Cortical firing dynamics during micro-arousals vary with sleep/wake history and micro-arousal duration
Source: Sci Rep. 2026 Apr 1;16:15391. doi: 10.1038/s41598-026-45192-y (PMC13183868; doi:10.1038/s41598-026-45192-y)
Supplement: Supplementary file 1 — Supplementary Material 1 [file 41598_2026_45192_MOESM1_ESM.pdf]

# Cortical firing dynamics during micro-arousals vary with sleep/wake history and micro-arousal duration

Natalie L. Hauglund[1,2,3,4]*, Lukas B. Krone[1,5,6], Martin Kahn[7], Cristina Blanco-Duque[1,7], Vladyslav V. Vyazovskiy[1,3,4]*

## Supplementary figures

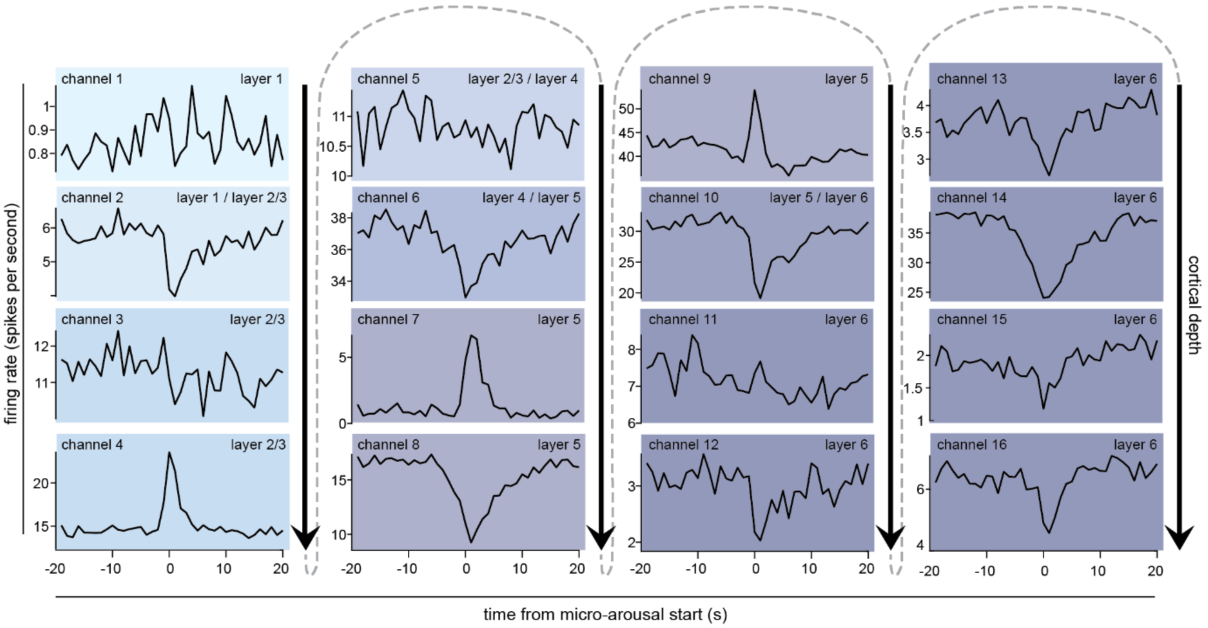


**Figure S1 – Example of firing rate across 16 channels**

Example firing rates around micro-arousals from one mouse. Each plot corresponds to the number of spikes per second for a single LFP channel. Colour coding shows increasing cortical depth. Note that channels exhibiting decreased and increased firing are spread across the cortical column and are not separated into specific layers.

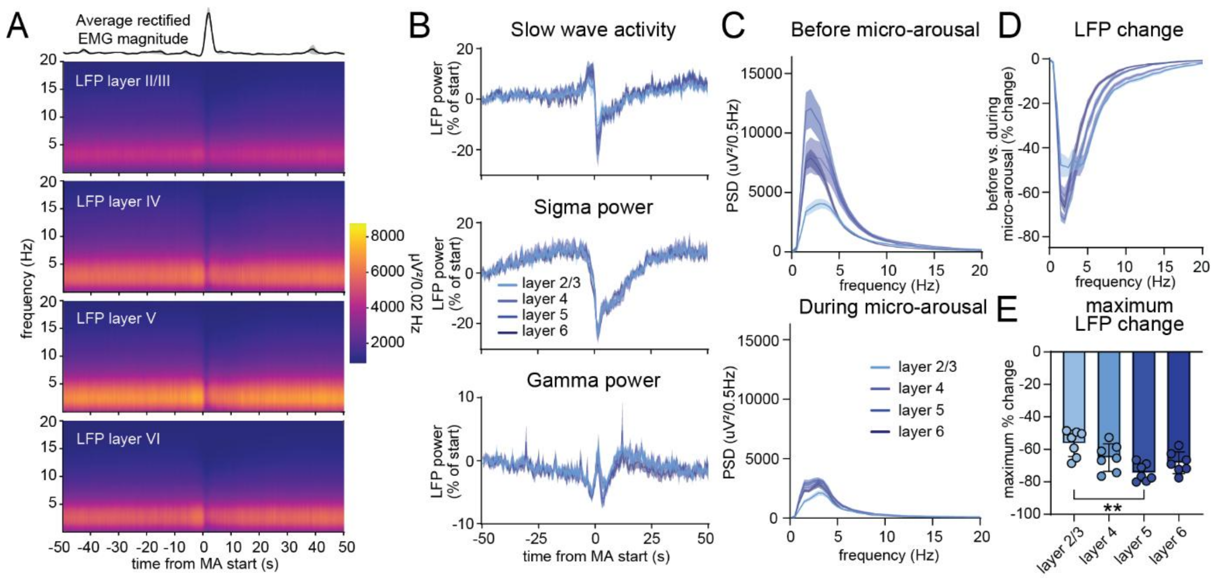


**Figure S2 – Micro-arousals elicit a decrease in LFP power throughout layers of the motor cortex**

(A) Top: mean rectified EMG trace and bottom: mean LFP spectrogram from layer 2/3, layer 4, layer 5, and layer 6. All are time-locked to the beginning of the micro-arousal start (n=7). (B) SWA, sigma and gamma power across layers 2/3, 4, 5, and 6 time-locked to the beginning of micro-arousals. Power is calculated as the percent change from 30-50 s before (n=7). (C) Power spectral densities for layers 2/3, 4, 5, and 6 before (t=-50 to -30 s) and during (t=0 to 2 s) micro-arousals (n=7). (D) Quantification of the percentage change between before and during micro-arousals for all layers (n=7). (E) Quantification of the maximum change from data shown in (D) (n=7, P=0.051 for layer 2/3 vs. layer 5, RM one-way ANOVA with Geisser-Greenhouse correction and Tukey's multiple comparison). **P=<0.01. Shaded area around line graphs are ±SEM. LFP = local field potential.

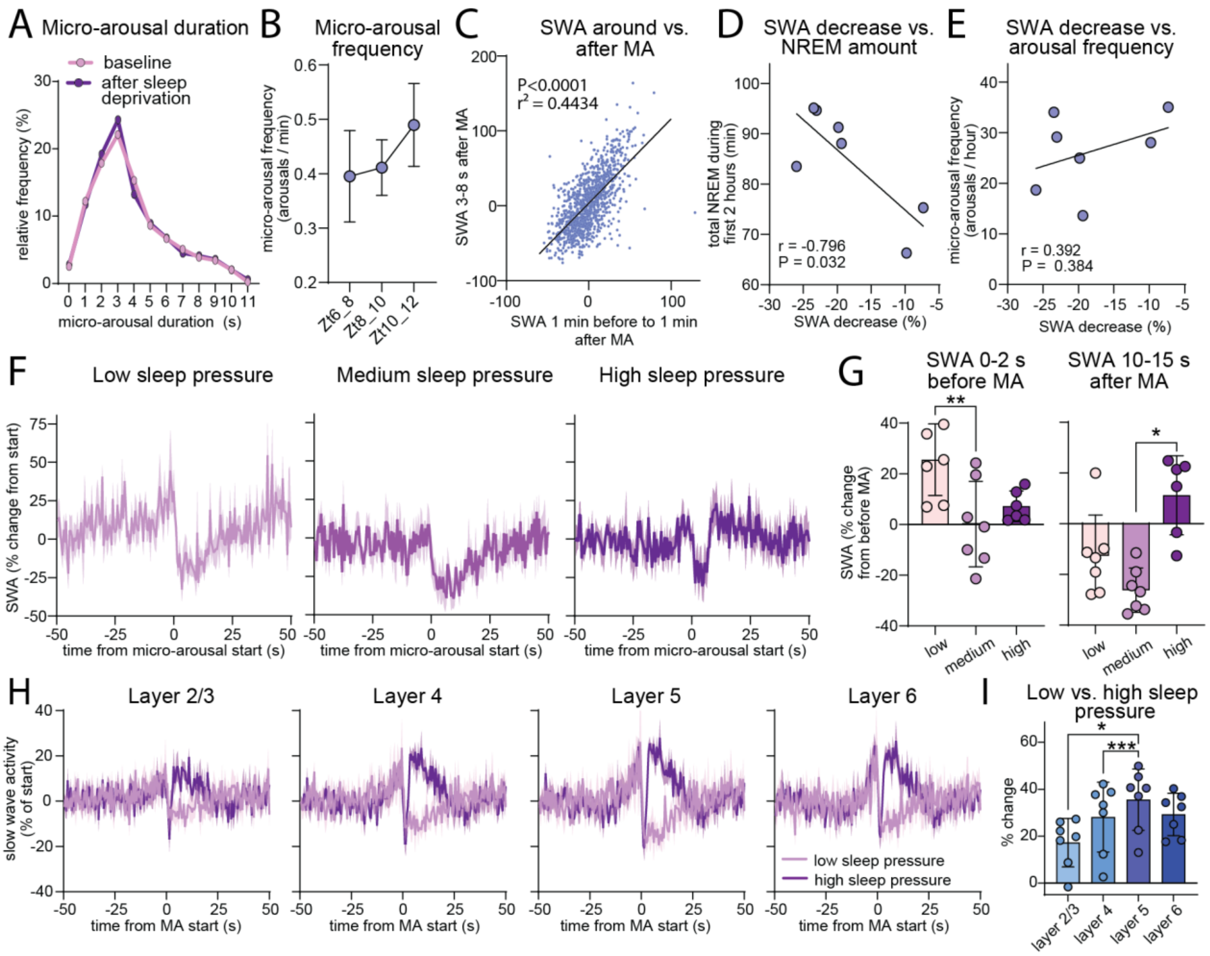


**Figure S3**

(A) Histogram showing the relative frequency distribution of micro-arousal durations during baseline and after sleep deprivation (n=7). (B) Quantification of the frequency of micro-arousals (duration <5 s) per minute in consecutive 2 hour bins following 6 hours sleep deprivation (n=7, P=0.379, RM one-way ANOVA with Geisser-Greenhouse correction). (C) SWA measured 1 minute before to 1 minute after each micro-arousal correlated to SWA 3-8 seconds after the same micro-arousal (Pearson correlation, 1162 micro-arousals from 7 mice during 18 hours after 6 hours sleep deprivation included, simple linear regression). (D-E) The decrease in average SWA amplitude from the first to the last NREM bout within the first two hours of recovery sleep after sleep deprivation correlated with the total amount of NREM sleep within the same period (D) and the average frequency of micro-arousals within the same period (E). (F) SWA expressed as percent change from 20-50 s before the micro-arousal during low (top panel), medium (middle panel) or high (bottom panel) sleep pressure (n=7). (G) Average SWA 0-2 s before the micro-arousal (top) and 5-10 s after the micro-arousal (bottom, n=7, RM two-way ANOVA with Geisser-Greenhouse correction and Tukey's multiple comparison). (H) SWA expressed as percent change from 20-50 s before the micro-arousal under low and high sleep pressure conditions for each cortical layer. (I) Quantification of the change in average SWA after the micro-arousal (5-10 s after the beginning of muscle activity) under low vs. high sleep pressure conditions (n=7, P=0.017 for layer 2/3 vs. layer 5 and P=0.0009 for layer 4 vs. layer 5, RM one-way ANOVA with Geisser-Greenhouse correction and Tukey's multiple comparison). MA = micro-arousal, SWA = SWA.
